# Robot-assisted Ivor Lewis Esophagectomy (RAILE): A review of surgical techniques and clinical outcomes

**DOI:** 10.3389/fsurg.2022.998282

**Published:** 2022-11-04

**Authors:** Tianzheng Shen, Yajie Zhang, Yuqin Cao, Chengqiang Li, Hecheng Li

**Affiliations:** Department of Thoracic Surgery, Ruijin Hospital, Shanghai Jiaotong University School of Medicine, Shanghai, China

**Keywords:** robotic surgery, Ivor Lewis esophagectomy, minimally invasive esophagectomy, esophageal cancer, clinical outcomes

## Abstract

In the past 20 years, robotic system has gradually found a place in esophagectomy which is a demanding procedure in the deep and narrow thoracic cavity containing crucial functional structures. Ivor Lewis esophagectomy (ILE) is a mainstream surgery type for esophagectomy and is widely accepted for its capability in lymphadenectomy and relatively mitigated trauma. As a minimally invasive technique, robot-assisted Ivor Lewis esophagectomy (RAILE) has been frequently compared with the video-assisted procedure and the traditional open procedure. However, high-quality evidence elucidating the advantages and drawbacks of RAILE is still lacking. In this article, we will review the surgical techniques, both short and long-term outcomes, the learning curve, and explicate the current progress and clinical efficacy of RAILE.

## Introduction

Esophageal cancer is one of the most life-threatening cancers with 544,076 patients dead in 2020 (1). The establishment of multimodal therapy effectively enhances surgical outcomes and long-term survival (2, 3). Currently, surgery remains the crucial and primary measure for the eradication of early and locally advanced esophageal cancer. The introduction of the da Vinci robotic system to esophagectomy, as a promising minimally invasive technique, aimed at reducing morbidity and mortality, improving long-term survival, and raising patients’ quality of life. It has been nearly 20 years since the first reported case of robot-assisted minimally invasive esophagectomy (RAMIE) case, and RAMIE is now frequently applied in high-volume esophageal surgery centers around the world (4–6). The robotic platform’s ergonomic design, tremor filtration, flexible articulation and three-dimensional vision, make it particularly suitable for a demanding esophagectomy which combines dissection and reconstruction in a deep dark cavity with important anatomical structures. Ivor Lewis procedure and McKeown procedure are both considered to be the mainstream surgery types nowadays, while transhiatal esophagectomy is less utilized for its skeptical ability in lymph node (LN) dissection (7). The theoretical advantages of robot-assisted Ivor Lewis esophagectomy (RAILE) have so far not been statistically defined. In this review, we summarize the existing publications to overview surgical techniques, short-term outcomes, long-term outcomes and the learning curve of RAILE, and offer our perspective on RAILE.

## Surgical techniques

In most high-volume centers, RAILE is performed with a da Vinci Surgical System (Intuitive Surgical, Inc., Sunnyvale, CA) and a four-arm technique. As many publications reported experience and details of different parts of RAILE (8–13), we generally summarize the well-accepted procedure. We propose several possible ways for the same step and the literature in which they are described in detail if they are currently performed with no significant increase in adverse events.

### Patient setup

**Figure 1:**
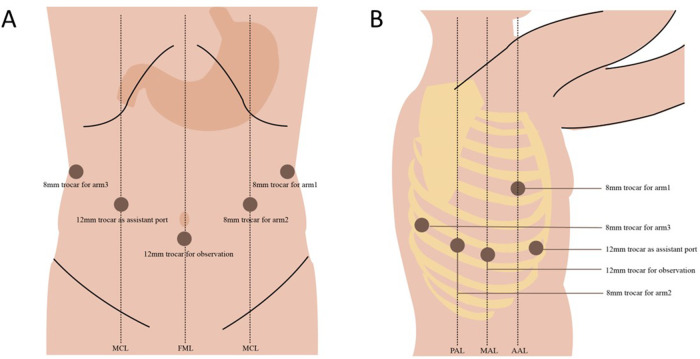
An example of patient positioning and trocar placement in our hospital. (**A**) Abdominal phase and (**B**) thoracic phase.

For the abdominal portion, the patient is positioned supine and in a 15°–25° reverse Trendelenburg position (with or without a ∼10° rotation to the right). Five trocars are most commonly placed (three for robotic arms, one for observation, and one as an assistant port). We normally do not apply a liver retractor but an additional subxiphoid incision may be formed to place a Nathanson liver retractor in certain institutions (9, 10). For the thoracic portion, the patient is placed in the left-lateral decubitus position in the thoracic phase with single-lung ventilation. Similarly, five trocars are usually placed. An example of trocar placement is demonstrated in Figure 1.

### Abdominal portion

**Figure 2:**
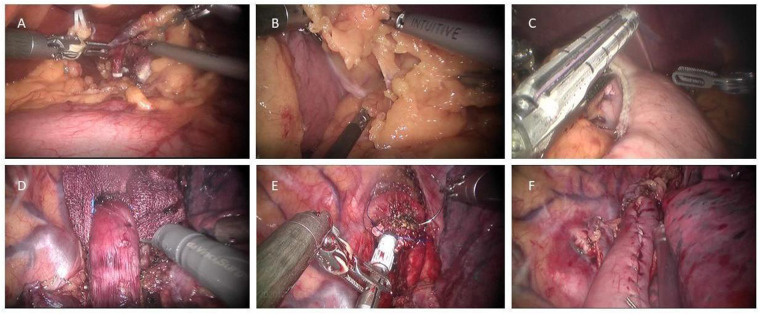
Key steps in robot-assisted Ivor Lewis esophagectomy. (**A**) After the lesser omentum was divided, the left gastric pedicle was exposed and divided with Hem-o-lock clips and a vessel sealer. (**B**) The gastrocolic ligament was divided toward the left gastroepiploic pedicle. (**C**) A 4–5 cm wide gastric conduit was formed toward the fundus with several fires of an Endostapler. (**D**) The esophagus was mobilized *en bloc* down to the gastroesophageal junction with dissection of all surrounding lymph node tissues. (**E**) The anvil of a 25-mm Premium Plus CEEA circular stapler was carefully inserted into the distal esophageal stump and fixated with two separate concentric purse-string sutures. (**F**) The form of a completed esophagogastric anastomosis.

The abdominal portion starts by retracting the liver, using either the purse-string suture and clips or a Nathanson retractor (10, 14). After the aberrant left gastric artery is evaluated, the hepatogastric ligament is dissected along the lesser curvature up to the right crus of the diaphragm. A D2 lymphadenectomy is then performed, covering LNs around the common hepatic artery, the left gastric artery, and the splenic artery. The left gastric vessels are ligated using Hem-o-lok Clip and the da Vinci Endowrist Vessel Sealer or Harmonic scalpel (Figure 2). As the lesser sac is now visualized by gently lifting the fundus, all colonic mesentery adhesions, residual ligaments, and short gastric arteries should be carefully dissected or ligated. The right crus of the diaphragm can be severed, which facilitates the opening of the gastrocolic ligament. The gastrocolic ligament is dissected along the greater curvature towards the spleen, from approximately 2 cm away from the gastroepiploic arcade. The left gastroepiploic vessels are divided, while the right ones are preserved. Kocherization of the duodenum is not routinely performed. At this point, the stomach has been completely mobilized (10). The abdominal portion can also begin with the greater curvature of the stomach and mobilize the stomach towards the crus of the diaphragm if preferred (12).

Thereafter, a gastric conduit measuring 4–5 cm is required to be formed. The conduit is developed from the pyloric antrum to the fundus along the greater curvature with several fires of an Endostapler with 45 mm/60 mm staplers. The apex of the conduit is connected to the inferior portion of the specimen by two interrupted silk sutures and marked with a stitch, allowing it to be lifted into the thoracic cavity without any torsion (14). Another possible option is to partially form the gastric tube in the abdominal cavity and then insert the circular stapler from the remnant stomach to alleviate microvascular damage and serve for end-to-end anastomosis (10).

Most institutions prefer to inject indocyanine green (ICG) intravenously to assess the perfusion of the conduit, which is reported to potentially decrease the risk of anastomotic leakage (15). Some institutions perform intramuscular Botox injections to the pylorus to improve early gastric emptying and prevent postoperative reflux (9, 16). However, these measures are not obligatory and must be further validated for effectiveness. Jejunostomy is regularly performed (usually 20–30 cm distally away from the ligament of Treitz), as the last step of the abdominal portion (9), to implant a feeding probe to ensure postoperative enteral feeding. However, the role of jejunostomy has not been concluded yet (17, 18).

### Thoracic portion

To begin the thoracic esophageal dissection, LNs are dissected around the right recurrent laryngeal nerve (RLN) and the arch of the azygos vein is divided. The esophagus is then mobilized *en bloc* down to the gastroesophageal junction, with all surrounding LNs in the periesophageal, periaortic, and subcarinal areas dissected. To avoid heat injury, periesophageal tissue should be meticulously cleared with special attention (11), using cutting devices such as Monopolar Cautery Hook, Harmonic Scalpel, and Bipolar Forceps. The thoracic duct is selectively clipped in some centers. After pulling up the conduit through the hiatus, the specimen and conduit are disconnected. The proximal esophagus is divided with robotic scissors 2–3 cm above the level of the azygos vein and sometimes to the thoracic inlet depending on tumor location. The specimen is removed through the wound protector and frozen section analysis is performed (This step is after anastomosis in case of the aforementioned partially formed gastric tube).

**Figure 3:**
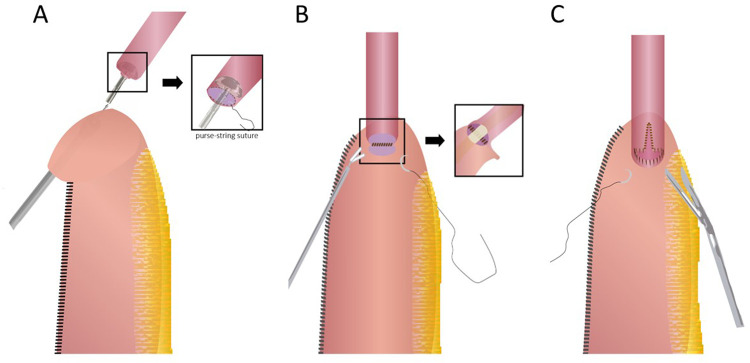
Three types of robot-assisted intrathoracic esophagogastric anastomosis. (**A**) Circular stapling technique; (**B**) fully robotic hand-sewn technique; (**C**) linear stapling technique with robotic hand-sewn closure of the stapler defect.

After the frozen section analysis, the esophagogastric reconstruction follows. There are three major methods used for reconstruction as described in the following paragraphs and Figure 3. The anastomosis can be finally reinforced with an omental wrap to prevent leakage (9, 12).

Circular stapled anastomosis: This is the most commonly used anastomosis technique in RAILE because of its relative reliability and simplicity. A 25/28/29 mm circular stapler anvil is inserted into the esophageal stump either transorally or transthoracically and fixated with two separate concentric purse-string sutures. The handle is then inserted into the conduit *via* an incision on the tip and pierced through the stomach wall on the greater curve side. After appropriately marrying the spike and anvil, the anastomosis is formed by firing. Finally, the proximal redundant conduit and gastrotomy are closed with an endostapler (12, 19).

Robotic hand-sewn anastomosis: Using a double-layer technique, the surgeon generally constructs the posterior and anterior walls of the anastomosis in order. The posterior seromuscular layer of the esophageal remnant is interruptedly sutured to the serosa on top or side of the gastric tube, followed by gastrotomy along the suture line and a running suture of the posterior mucosal layer. Then, the inner and outer layers of the anterior wall can be closed respectively with a single running suture and interrupted sutures or with interrupted sutures for both layers (11, 20).

Linear stapled anastomosis: The conduit and the esophageal remnant are partly overlapped. A small gastrotomy is performed about 4–5 cm below the tip of the conduit. The anvil parts are then placed separately in the conduit and the esophageal lumen, and an approximately 3 cm anastomosis is formed. The stapler defect is finally completed with a robotic hand-sewn technique, including the inner layer by running barbed sutures and the outer layer by interrupted sutures (13, 21, 22).[Fig F4]

**Figure 4 F4:**
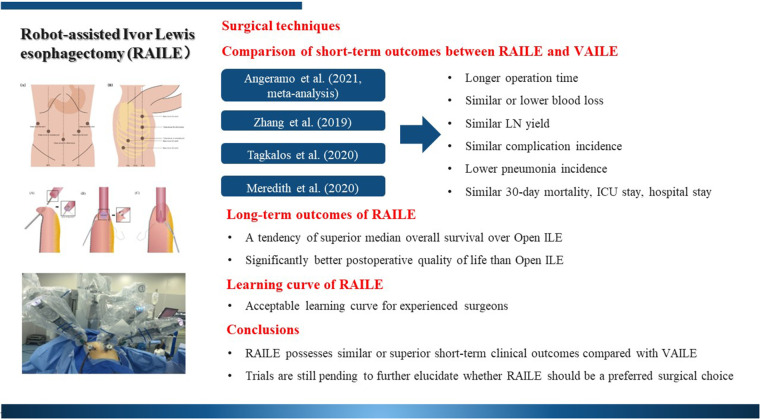
Graphical summary of robot-assisted Ivor Lewis esophagectomy and its clinical outcomes.

## Short-term outcomes

Several studies have demonstrated the feasibility and safety of robot-assisted esophagectomy *via* the Ivor Lewis procedure. As a promising technique of minimally invasive esophagectomy, thoracic surgeons are encouraged to compare it with the conventional laparoscopic-thoracoscopic one to discover latent benefits or defects. Angeramo et al. recently published the first meta-analysis of 5,275 video-assisted Ivor Lewis esophagectomy (VAILE) patients and 974 RAILE patients to statistically clarify the difference in surgical outcomes between these two minimally invasive approaches (23). An evidence-based comparison between RAILE and VAILE was also carried out as a subgroup analysis in the study conducted by Manigrasso et al. (24). However, heterogeneity existed between the included studies in terms of certain indicators, which impaired the credibility to some extent. The relevant studies on RAILE are illustrated in Table 1, categorized by their objectives.

**Table 1:** Patient characteristics and short-term outcomes of studies on robot-assisted Ivor Lewis esophagectomy (more than 50 patients).

Author	Objective	*N*	Patient characteristics					Intraoperative outcomes			Morbidity and mortality							Hospitalization	
			Median age	Sex, Male (%)	Histology, EAC (%)	Neoadjuvant therapy (%)	Anastomotic method	Op Time (min)	EBL (ml)	LNH	Anastomotic leaks (%)	Pneumonia (%)	Vocal cord paralysis (%)	Chylothorax (%)	Atrial fibrillation (%)	Overall morbidity (%)	In-hospital/ 30-day mortality (%)	ICU stay (day)	LOS (day)
Zhang Y (2019) (36)	RAMIE vs. VAMIE	76	62	78	0	0	CS/HS	304	200	19	9.2	6.6	6.6	1.3	6.6^#^	31.6	0.0 (90 d = 1.3)	NA	9.0
Tagkalos (2020) (35)	RAMIE vs. VAMIE	50	62	NA	NA	86	CS	383	331	27	12.0	12.0	NA	NA	NA	24.0	0.0 (90 d = 5.0)	1.0	12.0
Meredith (2020) (33)	RAMIE vs. VAMIE	144	66	79	NA	78	CS/HS	409	155	20	2.8	6.9	NA	NA	11.8*	23.6	NA (90 d = 1.4)	NA	9.0
Pointer (2020) (34)	RAILE vs. Open	350	66	83	87	81	CS	425	232	21	14.9	15.7	NA	1.7	23.7	74.3	2.6	NA	9.0
Kingma (2020) (32)	RAILE: multicenter experience	331	NA	NA	NA	NA	CS/HS/LS	400	100	28	19.6	23.3	1.0	4.8	15.0^#^	52.6	3.0	2.0	12.0
Grimminger (2021) (4)	RAILE: multicenter experience	175	61	85	81	82	CS/HS	385	28	28	10.3	16.0	NA	2.9	NA	32.0	1.1	NA	13.0
Egberts (2022) (29)	RAILE: multicenter experience	220	64	86	80	81	CS	425	200	25	13.2	19.5	NA	NA	NA	NA	NA (90 d = 3.6)	2.0	15.0
de la Fuente (2013) (28)	RAILE: single center experience	50	66	78	30	70	CS	445	146	20	2.0	10.0	NA	4.0	10.0	28.0	0.0	3.4	10.9
Hernandez (2013) (30)	RAILE: single center experience	52	65	79	88	67	CS	442	NA	19	1.9	9.6	NA	3.8	9.6	26.9	0.0	NA	NA
Cerfolio (2016) (27)	RAILE: single center experience	85	63	87	NA	75	LS	360	35	22	4.3	7.1	NA	5.9	7.1	36.5	3.5 (90 d = 10.6)	NA	8.0
Egberts (2017) (11)	RAILE: single center experience	75	66	68	96	79	CS/HS/LS	392	180	29	16.0	NA	NA	NA	NA	69.3	NA (90 d = 3.9)	NA	16.0
Zhang H (2019) (22)	RAILE: single center experience	77	62	88	18	21	CS/LS	350	111	21	6.5	9.0	6.5	1.3	1.3	39.0	0.0	1.0	12.3
Berlth (2020) (8)	RAILE: single center experience	100	66	84	NA	84	NA	NA	NA	NA	5.0	7.0	NA	NA	NA	NA	1.0	1.0	11.0
Kandagatla (2022) (31)	RAILE: single center experience	112	64	84	87	76	LS	357	65	19	8.5	10.4	1.9	NA	28.3*	NA	0.9 (90 d = 3.8)	NA	NA

RAMIE, robot-assisted minimally invasive esophagectomy; VAMIE, video-assisted minimally invasive esophagectomy; RAILE, robot-assisted Ivor Lewis esophagectomy; EBL, estimated blood loss; LNH, lymph node harvested; ICU, intensive care unit; LOS, length of hospital stay; 90 d, 90-day mortality; NA, not available.

^#^ Rate of total cardiac complication.

* Rate of arrhythmia.

### An overview of short-term outcomes of RAILE

Short-term outcomes of RAILE, as shown in Table 1, are generally satisfactory when compared with a modern global benchmark for outcomes associated with esophagectomy (25). The operation time ranges from 304 to 445 min and the median blood loss ranges from 28 to 331 ml. The average LN yield is between 19 and 29, which was theoretically adequate to retain precise *N* staging and guarantee long-term survival (26). Common complications related to esophagectomy include anastomotic leakage, pulmonary complications (such as pneumonia, respiratory failure, pleural effusion, and pneumothorax), vocal cord paralysis, severe cardiac complications (mainly arrhythmia), chylothorax, and wound infection. The anastomotic leak rate ranges from 1.9 to 19.6% (4, 8, 11, 22, 27–36). Despite using different anastomotic methods, some centers had leak incidences of less than 5%, suggesting the underlying importance of personal proficiency. The evidence to compare the surgical outcomes of these three methods is still limited (37). The prevalence of pneumonia ranges from 6.6% to 23.3% (benchmark: 13.4%). The frequency of chyle leaks ranges from 1.3% to 5.9% (benchmark: 4.7%). The records of cardiac complications were particularly inconsistent and showed an evident discrepancy in the incidence of atrial fibrillation ranging from 1.3% to 23.7% (benchmark: 14.5%). Vocal cord paralysis was barely recorded in the listed studies. As the documentation of complications and morbidity varied among the studies, results are recommended to be recorded in line with the Esophagectomy Complications Consensus Group (ECCG) agreements (38). Mortality is a more fundamental indicator to assess the quality of surgery. Most studies in Table 1 show uplifting results of 30-day mortality (0% in five studies, 0%–3% in four studies, 3%–5% in one study). However, it is worth mentioning that 90-day mortality can be observed as evidently higher than 30-day mortality, which is still concerned to be caused by tumor- and management-related factors (39). The 90-day mortality may be an appropriate and valuable indicator of quality after the complex RAILE surgery.

### Comparison between RAILE and VAILE

The mean operative time of RAILE was longer in all three studies comparing RAILE and VAILE (33, 35, 36). This was considered a disadvantage of RAILE because excessive prolongation of the operation (defined as over 422 min) raises the risk of pulmonary and infectious complications (40). However, we believe a factor that ought not to be neglected is the robotic repositioning time from the thoracic to the abdominal phase. Yang et al. applied a more scientific method of operation time calculation, i.e., excluding the period between the uninstallation of devices and the abdomen incision. In this scenario, they obtained an unexpected result that a significantly shorter operation time was taken in RAMIE (*p* < 0.001) (41). Angeramo's meta-analysis showed lower intraoperative estimated blood loss (EBL) in RAILE (144.3 ml vs. 213.6 ml, *p* = 0.006) (23). Of the three studies independently comparing RAILE and VAILE, one reported significantly higher LN yield conducted by RAILE (33), one showed a trend in favor of RAILE (35), and one reported no significant difference (36), suggesting better or similar LN yield in RAILE.

### Comparison between RAILE and Open ILE

Comparison between RAILE and Open ILE has been relatively scarce, mainly because of its minimally invasive nature. As certain benefits of VAILE over Open ILE have been explicit (42–44), once we understand that RAILE and VAILE have similar or even better postoperative outcomes, we can assume that RAILE would possess benefits over Open ILE. Na et al. found that RAILE led to comparable complication incidence, lower rate of major complications and decreased LOS (13 vs. 15 days, *p* = 0.03) than Open ILE (45). Meanwhile, RAILE showed stronger capability in LN retrieval (42.8 vs. 35.3, *p* < 0.01). In another existing study in which 222 RAMIE were matched 1 : 1 to the Open ILE control, RAILE demonstrated shortened LOS (9 vs. 10 days, *p* = 0.01), lower reoperation rates (2.3 vs. 12.2%, *p* = 0.001), and extended operative time (427 vs. 311 min, *p* = 0.001) (34). An RCT has already demonstrated fewer surgery-related complications and better postoperative quality of life brought by RAMIE instead of open esophagectomy in the McKeown procedure (46). A similar trial in the Ivor Lewis procedure is still pending.

## Long-term outcomes

### Overall survival and recurrence-free survival of RAILE

Both 5-year overall survival (OS) and 5-year recurrence-free survival (RFS) are still fundamental metrics to evaluate the effect of RAILE (Table 2). Na et al. reported in their propensity score-matching (PSM) analysis that 5-year OS was significantly higher in the RAILE group (75.1% vs. 57.9%, *p *= 0.02), while 5-year RFS was comparable (68.8% vs. 54.7%, *p *= 0.15) (45). They additionally noted that the 5-year rate of RFS regarding regional LN recurrence was higher in the RAILE group, with local and distal recurrence being detected with no positive finding. Another two relevant studies were carried out under hybrid RAILE, in both of which the transthoracic part was performed by a robotic platform (31, 34). Kandagatla found a 5-year OS of 49.4% and a 5-year RFS of 44.0% in patients undergoing the RAILE procedure (31). Although the results seem to be inferior to those by Na et al., it is explicable because of the more advanced pathologic staging in the patient population. Meanwhile, 343 RAILE patients being matched to the Open ILE cohort in the PSM analysis by Pointer showed a tendency of superior median overall survival (63 vs. 53 months, *p* = 0.13) (34). Such superiority in long-term survival can be possibly explained by the elevated capacity of LN dissection of RAILE over Open ILE (47). A recent population-based study analyzing the long-term effects of RAMIE revealed that RAMIE brought us significantly better overall survival over OE [hazard ratio (HR) 0.81, 95% CI: 0.68–0.96, *p* = 0.017], and no difference was detected between RAMIE and VAMIE (HR 0.99, 95% CI: 0.90–1.09, *p* = 0.8) (5).

**Table 2:** Patient characteristics and long-term outcomes of studies on robot-assisted Ivor Lewis esophagectomy.

Author	*N*	Patient characteristics					Survival outcomes				
		Median age	Sex, Male (%)	Histology, EAC (%)	Neoadjuvant therapy (%)	Median positive LN	Median retrieved LN	Median follow-up period (m)	Median OS (m)	5-year OS (%)	5-year RFS (%)
Pointer (2020) (34)	350	66	83	87	81	0.7	22.4	NA	63.3	NA	NA
Na (2021) (45)	136	65	90	0	26	1.4	42.8	31.8	NA	75.1	68.8
Kandagatla (2022) (31)	112	64	84	87	76	0	19	NA	NA	49.4	44.0

5-year OS, 5-year overall survival; 5-year RFS, 5-year recurrence-free survival.

### An elevated quality of life brought by RAILE

Patients who underwent RAILE procedures also tend to have a better quality of life than those who underwent Open ILE. This is utterly important in our view because creating a maximum quality of life for patients with esophageal cancer within their expected limited lifespan aligns with the humanitarian imperative. Mahdorn et al. investigated self-perception and quality of life of postoperative RAILE patients with the European Organization for Research and Treatment of Cancer (EORTC) Quality of Life Questionnaire Core-30 (QLQ-C30) questionnaire at 4 and 18 months after surgery, respectively (48). RAILE patients reported better global health status after 4 months than Open ILE patients, with less fatigue, nausea, vomiting, pain, dyspnea, appetite loss, and diarrhea, as well as better function in all dimensions. After a longer period of 18 months, RAILE patients were reported to have significantly better recovery, with the symptoms further alleviated, functions further reestablished and some even returned to the level of the general population (48).

## Learning curve of RAILE for thoracic surgeons

To optimize the surgical outcomes of RAILE, thoracic surgeons will have to experience a learning curve. Our group lately presented our results of the learning curve of RAILE within 124 consecutive patients by risk-adjusted-cumulative sum analysis (49). We found that 51 cases were the baseline to achieve acceptable surgical outcomes and proficiency and 73 cases were needed to further make a difference in blood loss and LN yield (49). In comparison, the 22nd case represented the inflection point, resulting in less blood loss, shorter operative time, and a lower rate of postoperative pneumonia in German multicenter research (29). We thus speculate the Upper GI International Robotic Association (URIGA) structured training pathway implemented in Germany may be a crucial factor. Several earlier studies agreed with the reduction of operation time after approximately 20 cases without reporting perioperative outcomes (19, 30). Most of the RAILE articles in the past 20 years, as shown in Table 1, are inevitably influenced by the effect of the learning curve. Future publications may better illustrate the strength of RAILE, with more senior surgeons successfully surpassing the learning curve and obtaining proficiency.

## Perspective

Since its introduction into esophagectomy, the robotic platform has developed and thrived in the field of esophageal surgery (50, 51). With more advantages of RAILE being confirmed, it may develop into a popular surgical option for patients in the future. First, as a robotic platform provides us with high-quality images and makes stable and flexible movements in the thoracic cavity (51), it has noninferior clinical results to VAILE. Second, RAILE patients have similar survival and elevated quality of life after the operation. Meanwhile, the learning curve of RAILE is acceptable. Demerits of RAILE mainly point toward the cost issue and the relatively inferior outcomes in low-volume centers (52). Soon, the ROBOT-2 Trial (NCT04306458) will be the first study to directly compare RAILE with VAILE in middle/distal esophageal or GEJ adenocarcinoma, with LN dissection as the primary endpoint (53). RAILE Trial (NCT03140189) conducted by our center, as a prospective, single-arm trial (phase II) collecting major complication rates and OS, recently finished patient follow-ups and the results will soon be posted. The trials above may further elucidate whether RAILE should be a preferred surgical option.

Besides, the theoretical survival benefit of three-field lymphadenectomy turned out to be limited and may add postoperative complication risks in esophageal cancer patients with lower tumor locations in recent studies. Koterazawa found that three-field lymphadenectomy resulted in a higher incidence of RLN palsy (14% vs. 26%, *p* = 0.046) without elevating 5-year OS (54). The research article published by Li et al. in 2020 strongly indicated that in middle and lower esophageal cancer, there was no significant difference in OS and disease-free survival (DFS), as well as in postoperative complications, between patients receiving three-field lymphadenectomy and two-field lymphadenectomy (55, 56). These clues suggest that RAILE could be more widely accepted in the future when it is oncologically feasible.

In conclusion, RAILE is an effective minimally invasive technique to ensure the feasibility and safety of esophagectomy, with similar or superior clinical outcomes compared with VAILE (Figure 4). With more studies aiming at uncovering the latent advantages, RAILE is likely to have a broader and more mature application.
